# Role of the viral protein UL24 in nucleolar modifications induced by herpes simplex virus 1

**DOI:** 10.1186/1753-6561-5-S1-P102

**Published:** 2011-01-10

**Authors:** Angela Pearson, Amélie Bourget, Nawel Ben Abdeljelil, Maria H Lymberopoulos

**Affiliations:** 1 INRS-Institut Armand-Frappier, INRS, Laval, Québec, Canada H7V 1B7

## 

Herpes simplex virus 1 (HSV-1) infection induces multiple modifications to nucleoli. We and others have observed the redistribution of nucleolar proteins such as nucleolin, fibrillarin, B23 (nucleophosmin), upstream binding factor (UBF) and RNA polymerase I (RNAPI) during HSV-1 infection. UL24 is one of a limited number of HSV-1 proteins that localizes to nucleoli. In a murine model of ocular HSV-1 infection, viruses that do not express the UL24 protein exhibit reduced viral titers in the eye and in trigeminal ganglia, and a reduction in clinical signs of disease. UL24 is conserved among *Herpesviridae*. Specifically, the N-terminal region of the protein contains several stretches of highly conserved residues. Bioinformatics studies have identified an endonuclease motif in the N-terminal portion of UL24, although as yet, no nuclease activity has been demonstrated for the protein. Previously, we discovered that UL24 transiently localizes to nucleoli during infection, and is both necessary and sufficient to induce the dispersal of nucleolin from nucleoli throughout the nucleus. In contrast, the redistribution of fibrillarin is a UL24-independent event. Moreover, the largest subunit of RNAPI and the transcription factor UBF are redistributed to viral replication compartments and these effects are also UL24-independent. We recently investigated whether UL24 was implicated in other HSV-1-induced nucleolar modifications. We discovered that UL24 was involved in the redistribution of B23 during infection. Furthermore, expression of UL24 in the absence of other viral proteins was sufficient to induce the relocalisation of B23. Similar to what we found for nucleolin, the conserved N-terminal portion of UL24 was important for its effect on B23 redistribution. The impact of the mutant vUL24-E99A/K101A, in which the endonuclease motif has been altered, and of vUL24-G121A, which harbours an amino acid substitution outside of this motif, were tested. While the G121A mutation had only a modest effect on the ability the virus to induce the relocalisation of B23, the E99A/K101A mutation appeared to abolish this function. Interestingly, the E99A/K101A mutation was previously shown to have a large impact on viral pathogenesis in mice. We found that the effect of HSV-1 on the redistribution of B23 was similar in non-immortalized cells such as human foreskin fibroblasts, as in immortalized cell lines such as vero cells. The similarities with regard to the impact of UL24 on the spatial distribution of nucleolin and B23 suggest that these effects may be related to the same function of UL24 during HSV-1 infection.

